# Tsunami, post-tsunami malaria situation in Nancowry group of islands, Nicobar district, Andaman and Nicobar Islands

**Published:** 2011-01

**Authors:** Sathya Prakash Manimunda, Attayoor Purushottaman Sugunan, Wajid Ali Sha, Shiv Shankar Singh, Ananganallur Nagarajan Shriram, Paluru Vijayachari

**Affiliations:** *Regional Medical Research Centre (ICMR), Port Blair, Andaman and Nicobar Islands, India*; * *GB Pant General Hospital (Directorate of Health Services), Port Blair, Andaman and Nicobar Islands, India*

**Keywords:** Andaman and Nicobar Islands, construction, labour migration, malaria, *Plasmodium falciparum*, tsunami

## Abstract

**Background & objectives:**

Due to tsunami in 2004 a large proportion of population in Nicobar group of Islands become homeless, and in 2006 large scale labour migration took place to construct the houses. In 2008, a significant increase in malaria incidence was observed in this area. Therefore, in March 2008, the situation of malaria was assessed in Nancowry Islands in Nicobar District to study the reasons for the observed upsurge in the number of cases, and to suggest public health measures to control the infection.

**Methods:**

The methods included a retrospective analysis of long term trend in the behaviour of malaria over the years from 2001 to 2008, analysis of the acute malaria situation, and rapid fever and malaria parasitemia survey along with environmental component. Mass radical therapy (MRT) and post-intervention parasitemia survey were carried out. The malaria situation in the aftermath of MRT was analysed.

**Results:**

During the post tsunami year (2005) there was a large increase in the incidence of malaria and this trend continued till 2008. The percentage of Plasmodium falciparum increased from 23 to 53 per cent from 2006 to 2007 that coincides with the labour influx from mainland. The study showed that Nancowry was highly endemic, with high transmission setting, and high risk area for malaria. Though, more number of migrant labourers suffered fever (75 vs 20%) and sought malaria treatment over past month but parasitemia survey showed higher point prevalence of malaria among native tribes (7.4 vs 6.5%). Post-MRT, there was a decline in the occurrence of malaria, though it did not last long.

**Interpretation & conclusions:**

The study findings suggest that the migrant workers hailing from non-endemic or moderately endemic settings became victims of malaria in epidemic proportion in high endemic and high transmission setting. To find out the reasons for deterioration of malaria situation at Nancowry in the aftermath of tsunami requires further research.

About half the world’s population (3.3 billion) live in areas that have some risk of malaria transmission and one fifth (1.2 million) live in areas with a high risk of malaria (more than 1 reported case per 1000 population per year). India had an estimated 10.6 million malaria cases in 2006 that account for approximately 60 per cent of cases in the WHO South- East Asia region^1^.

Andaman and Nicobar Islands, a union territory of India, has historically been known for high malaria transmission^2^. Nicobar group of islands lie 143 miles south of Port Blair, capital of archipelago^3^. During the recent past the malaria situation in Nicobar group of islands is worse in comparison with Andaman group of islands^4^. The tropical climate prevailing throughout the year provides ideal environment for mosquito proliferation and breeding in Nicobar group of islands^5^. Entire land mass in the islands has network of creeks which results in an ideal brackish water habitats for the breeding of *Anopheles sundaicus*, the predominant vector of malaria in Andaman and Nicobar Islands^6^–^9^.

December 26, 2004 earthquake and subsequent tsunami caused inundation of sea water in to inland up to 1000 m^10^. The intense geological activity resulted in raising of north Andaman between 0.5 and 0.8 m, whereas the south Andaman and Nicobar experienced subduction of similar intensity^10^,^11^. Natural disaster, particularly meteorological events such as cyclones, hurricanes, flooding, and tsunami can affect vector breeding sites and vector-borne disease transmission^12^. The crowding of infected and susceptible hosts, a weakened public health infrastructure and interruption of ongoing control programmes are all risk factors for vector- borne disease transmission^13^,^14^.

In the aftermath of tsunami the spread and risk of malaria in the affected places was anticipated by experts^15^. As anticipated, in 2005, there was an increase of 1243 per cent in annual parasite incidence (API) (from 0.64 to 8.15) for Andaman District and it was almost 816 per cent (from 8.0 to 73.27) for Nicobar District. In the subsequent years after 2005, in the Nicobar group of Islands the malaria situation differed from island to island. A control was achieved in Car Nicobar but it deteriorated in Nancowry^16^.

Due to tsunami entire 45000 population of Nicobar group of Islands rendered homeless. Hence, from mid 2006 onwards large scale labour migrations from the districts of West Bengal started and were engaged in the construction of permanent shelters for the tsunami affected population. Population mobility due to various economic pressures and necessities is incriminated as one of the reason for complicating, as well changing the epidemiology of malaria^17^–^21^.

In March 2008, a large increase in the occurrence of malaria was observed in Nancowry group of islands, Nicobar District. In response to this, a team from the Centre was deputed to these islands to assess the situation of malaria. This study was carried out to confirm the diagnosis and the increase in the incidence of malaria as well to identify the possible causes of this increase, and to suggest measures for control of malaria in Nancowry group of islands.

## Material & Methods

### 

#### Study area:

The study was carried out in the jurisdiction of Community Health Centre (CHC), Nancowry, situated at Kamorta. Nancowry is the central group of islands in Nicobar District with hilly terrain covered with grass, forming undulating meadows. It consists of 12 villages, many are islands connected only by sea route. The population of the CHC area in 2008 was 4875, all aboriginal Nicobarese tribe^3^. Though the 2004 earthquake and subsequent tsunami damaged the dwellings of aborigines but loss of life was less than 20 persons. From mid 2006 onwards around 600 migrant male labourers from the districts of West Bengal were engaged in construction of permanent shelters in these islands. There was constant movement of labourers between these islands and main land India since mid 2006 with fresh batch replacing the one which went back. No batch stayed more than four months. The main reason for return was illness with fever presumed to be malaria. During February 2008 a fresh contingent of around 300 labourers arrived to Nancowry. During the time of study there were around 600 migrant male labourers aged between 18 to 55 yr. They were living in 6 of 12 villages of Nancowry in labour camps.

The methods of investigations included:-

1. *A retrospective analysis of long-term trend in the behaviour of malaria over the years from 2001*: Number of smears examined and positive cases over a period of seven years was collected from malaria surveillance data (Sur SK, personal communication). The number of tribal population during the years 2001-2008 was obtained from the register maintained in CHC, Nancowry, and migrant labourers were obtained from the Office of the Assistant Commissioner (AC), Nancowry. Monthly parasite incidence (MPI) and annual parasite incidence (API) were plotted to understand the long term and seasonal trend in the occurrence of malaria. The break-up of proportion of blood smears examined by active and passive surveillance was calculated. The statistical significance in change of API over previous year, the change in proportion of *Plasmodium falciparum* (*Pf*) cases over previous year, and the change in annual blood examination rate (ABER) over previous year (all from 2001-2007) was tested by χ^2^ test.
2. *Analysis of the acute malaria situation*: The daily number of confirmed cases of malaria detected from March 1, 2008 was compared with that during the same months for the previous year. The number of confirmed cases of malaria attending CHC, Nancowry, and the demographic details of patients such as age, sex, ethnicity, *etc*. were collected for a period of past three months. Incidence of acute malaria infections (percentage by age) among stable indigenous Nicobarese population from January 2008 to March 2008 was calculated to know the endemicity level of malaria at Nancowry^22^. Monthly incidence of malaria for preceding three months was calculated from the hospital registry of CHC, Nancowry; among native tribes and migrant labourers separately and the statistical significance in difference was tested by χ^2^ test. Slide positivity rate (SPR) was calculated for past three years and past three months separately to analyze the parasite buildup in the community^23^,^24^.
3. *Rapid fever and malaria parasitemia survey*: A cross-sectional survey was carried out in March 2008 to estimate the point prevalence of fever and malaria among indigenous tribal population and migrant labourers. This was carried out in six villages where 2743 tribal population and almost all 600 odd migrant labourers lived, prior to mass radical therapy (MRT). In these six villages every third household was surveyed from the list maintained in CHC, Nancowry, and first household being a random choice. Similarly every third labourer was chosen from each camp first being a random choice, from a list maintained in Office of AC, Nancowry. Informed consent from all the subjects was taken. Information about the history of fever during past month and bed net usage was also sought.
  The difference in point prevalence of fever and slide positivity among indigenous tribes and migrant labourers, the difference in slide positivity of young tribal children (aged < 10 yr) and elder tribal, as well the difference in proportion of history of having fever over past month among indigenous tribes and migrant labourers were tested by χ^2^ test.
4. *Environmental observation*: An observation was made for the possible breeding sites of vector mosquito.
5. *Mass intervention*: MRT was carried out in all the 12 villages and entire 4875 tribal people and 600 odd migrant labourers by Health Services, 600 mg of chloroquine and 45 mg of primaquine was administered from April 05-07, 2008. For children age appropriate dose was given and pregnant mothers were exempted. It was supervised and directly observed treatment (DOT) given and informed consent from all the subjects was taken.
6. *Post intervention malaria parasitemia survey*: Cross-sectional survey for malaria parasitaemia was repeated 8-10 days after initiating MRT. Information on compliance with MRT was also obtained by interviewing the subjects. This survey was carried out in the same six villages and among same house-holds and subjects. Every second household from the previously surveyed households were chosen, first being a random choice and among migrant labourers every second labourer was chosen from the previously surveyed labourers, first being random choice. Informed consent from all the subjects was taken.
7. *The study of malaria situation post-intervention*: Day wise occurrence of malaria in April 2008 was compared with the same in April 2007. The MPI in the subsequent months following MRT was compared with the same months of previous year. The statistical significance was tested by χ^2^ test. The malaria situation at Nancowry during the year 2008 was analysed by calculating API, percentage (%) of *Pf* and ABER. The statistical significance of change in API, percentage of *Pf*, and ABER over previous year was tested by χ^2^ test.

## Results

**Fig. 1:**
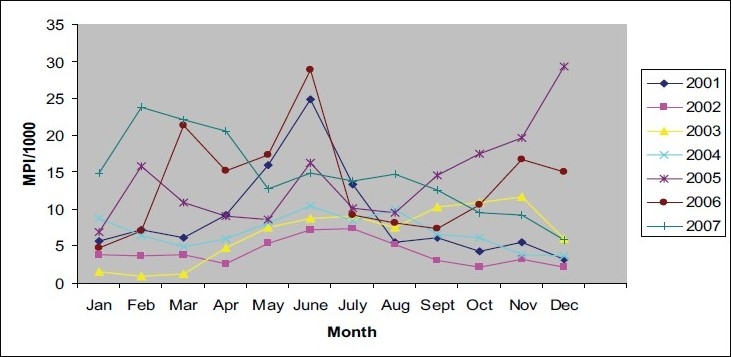
Monthly parasite incidence (MPI) at CHC, Nancowry, 2001-2007.

Fig. 1 shows the MPI of confirmed cases of malaria at CHC, Nancowry for 2001-2007. Malaria occured in all months. There was a peak in the month of June in all years. There was a large increase in API during 2005 (post tsunami year) and percentage of *Pf* during 2007 (Table I). Almost 2/3^rd^ of the smears examined were by active surveillance.

**Table I:** Annual parasite incidence (API), percentage of *P. falciparum* (*Pf*) diagnosed, and trend in malaria surveillance at CHC, Nancowry from 2001-2007

Year (population)	Smears examined, No. (%)			Total confirmed cases	API/1000	Change in API over previous year (%)	% of *Pf*	Change in % of *pf* over previous year (%)	ABER
	Active	Passive	Total							
2001(4573)	3706 (64)	2084 (36)	5790	490	107.2		23		126.6
2002 (4614)	4072 (70)	1745 (30)	5817	230	49.9**	-53	14**	-39	126.1
2003 (4656)	3340 (58)	2419 (42)	5759	373	80.1**	+61	20*	+43	123.7*
2004 (4699)	3584 (65)	1930 (35)	5514	391	83.2	+4	15	-25	117.3**
2005 (4742)	5920 (70)	2537 (30)	8457	798	168.3**	+102	22*	+47	178.3**
2006 (4786+600)	3917 (60)	2611 (40)	6528	815	151.3*	-10	23	+5	121.2**
2007 (4830+600)	8299 (70)	3556 (30)	11855	949	174.8**	+16	53**	+130	218.32**

(API=Total confirmed cases in a year/total population×1000); ABER annual blood examination rate (ABER, Total blood smears examined in a year/total population×100);

P*<0.05

** <0.01 compared to previous year

**Fig. 2:**
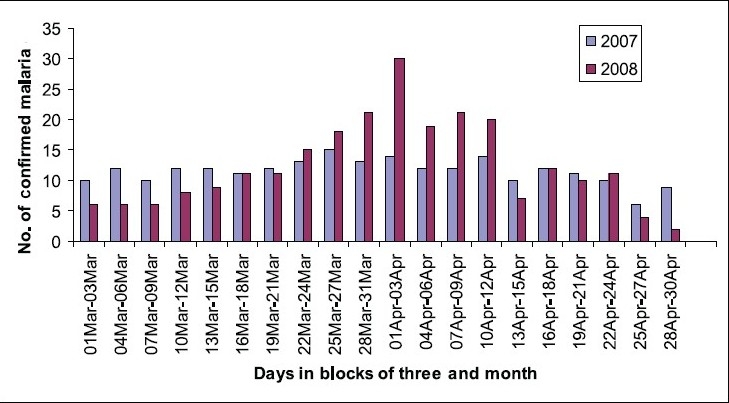
Occurrence of malaria March 2007 versus March 2008 and April 2007 versus April 2008 at CHC, Nancowry.

There was steady increase in the occurrence of malaria during the last week of March 2008 when compared with March 2007 of the same period (Fig. 2).

**Fig. 3:**
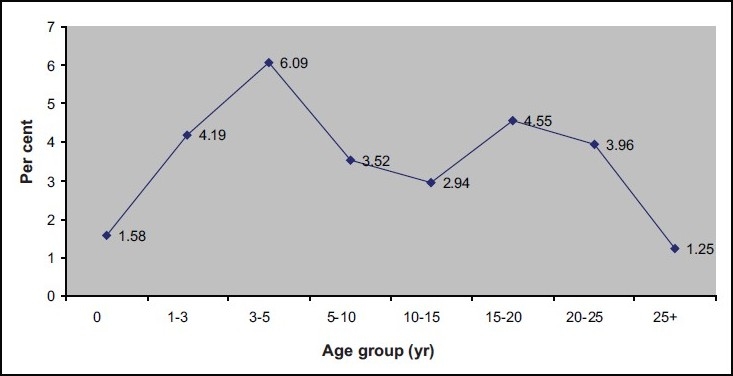
Incidence of acute malaria infections (percentage by age) among stable indigenous Nicobarese population (January-March 2008).

Incidence of acute malaria infections (percentage by age) among the Nicobarese showed high age specific incidence in late infancy or early childhood and among adolescents less and still less in adults indicative of high endemicity (Fig. 3).

It was found that the monthly incidence of malaria during January, February and March 2008 among migrant labourers was significantly higher (*P*,<0.01) than that among the Nicobarese (Table II).

**Table II:** Monthly incidence of malaria among Nicobarese and migrant labourers at CHC, Nancowry from January-March 2008 (from in- and out-patient register)

	Malaria incidence, 2008 (%)		
Population	January	February	March
Nicobarese tribe	0.84 (41/4875)	0.92 (45/4875)	0.73 (36/4875)
Migrant labourers	4.5* (27/600)	10.83* (65/600)	12.5* (75/600)

* *P*<0.01 compared to Nicobarese tribe

The slide positivity rate (SPR) for the year 2008 till April 4 was 8.60 per cent (277/3218). The SPRs for preceding three years, *i.e*. 2007, 2006, and 2005 were 8.0 per cent (949/11855), 12.48 per cent (815/6528), and 9.43 per cent (798/8457) respectively (Mean: 9.97). There was no increase in the SPR till April 4^th^ 2008 in comparison with preceding three years.

The SPR for initial four days of April 2008 was 20 per cent (70/350). The SPR for preceding three months, *i.e*. January, February, and March 2008 was 4.94, 7.90, and 8.88 respectively (mean ± SD: 7.24 ± 2.05). The SPR during the first week of April 2008 exceeded by more than three times of the standard deviation observed in SPR of the preceding 3 months of the same year.

Almost 30 per cent (823/2743) of the Nicobarese tribes and 30 per cent (217/600) of the migrant labourers were included in the rapid fever and malaria parasitemia survey. Among aborigines 51 per cent (420/823) were males and 49 per cent (403/823) were females and around 8 per cent (66/823) were aged <5 yr and almost 16 per cent (130/823) were aged <10 yr. This figure was comparable to the gender and age composition of CHC, Nancowry. The migrant labourers were aged between 18 and 55 yr and all were males.

Overall point prevalence of fever was 12.0 per cent [95% Confidence Interval (CI):10.1, 14.2] (125/1040). The point prevalence of fever among persons belonging to indigenous tribe was 10.3 per cent (95% CI: 8.3, 12.6) (85/823). Among migrant labourers it was 18.4 per cent (95% CI: 13.5, 24.2) (40/217). This difference in point prevalence of fever was statistically significant (*P*<0.01).

The overall slide positivity rate was 7.2 per cent (95% CI: 5.7, 8.9) (75/1040). The percentage of Pf was 56 per cent (42/75). The slide positivity rate among tribal was 7.4 per cent (95% CI: 5.7, 9.4) (61/823). The positivity rate among tribal children aged < 10 yr was 13 per cent (17/130), whereas that among older tribal people was 6.3 per cent (44/693). The difference was statistically significant (*P*<0.01). The slide positivity rate among migrant labourers was 6.5 per cent (95% CI: 3.6, 10.6) (14/217). The difference in the slide positivity rate among Nicobarese tribe (7.4%) and among migrant labourers (6.5%) was statically not significant. Out of the 40 tribal children aged <10 yr who had fever during the time of cross-sectional survey, 17 (42.5%) tested positive for malarial parasite.

Almost 10 per cent (6/61) of the tribal adults had asymptomatic parasitemia and none among migrant labourers. Over the past one month almost 75 per cent (163/217) of the migrant labourers and 20 per cent (165/823) aborigines reported having suffered fever. The difference was statistically significant (*P*<0.01). Bed net usage among the population of the island was almost 100 per cent (1039/1040). Impregnated bed net was not used by migrant labourers.

The heavy rain fall during the study period helped in better understanding of possible ecological reasons aiding vector proliferation. Four important observations were made: (*i*) The contact of sea water to the inland has increased due to geographic tilt. (*ii*) The habitat of tribal was shifted from sea shore to interior jungles to open temporary shelters following tsunami and this might have increased the chances of getting bitten by vector mosquito. (*iii*) The ongoing large scale construction activity and digging of land has created numerous artificial habitats for the breeding of mosquitoes. (*iv*) In the low lying areas the inundation of sea water and subsequent trapping has created numerous pools of water and the monsoon streams have become more stagnant in the aftermath of tsunami.

The reported coverage of MRT was 90 per cent among aborigines and 96 per cent among migrant labourers. Post MRT the peripheral blood smear of 500 subjects in the same six villages were taken and examined for malarial parasites. This included 15 per cent (410/2743) of the aborigines and 15 per cent (90/600) of the migrant labourers. Only two subjects of native tribe tested positive for malarial parasite and both were *Pf* species. One did not receive MRT and the other was a child aged 3 yr. None of the migrant labourers were positive for malaria parasite. Almost 5 per cent (4/90) of the migrant labourers and 7 per cent (30/410) of the native tribes missed MRT for one or the other reasons.

There was a fall in occurrence of malaria in April 2008 at CHC, Nancowry following the administration of MRT (Fig. 2). Table III shows the occurrence of malaria cases at CHC, Nancowry, during the subsequent months of 2008 following MRT and the comparison between the occurrences of malaria cases during the same months in 2007. The decrease in the occurrence of malaria was statistically significant (*P*<0.01) only in the month of June, 2008.

**Table III:** Mass radical therapy (MRT) and the occurrence of malaria at Nancowry during subsequent months of 2008

Month	Number of malaria cases		*P*
	2007 (MPI/1000)	2008 (MPI/1000)	
May	69 (12.7)	53 (9.7)	0.14
June	81 (14.9)	51 (9.3)	< 0.01
July	75 (13.8)	58 (10.6)	0.13
August	80 (14.7)	83 (15.2)	0.81
September	68 (12.5)	76 (13.9)	0.50
October	52 (9.6)	123 (22.5)	< 0.01
November	50 (9.2)	128 (23.4)	< 0.01
December	32 (5.9)	134 (24.5)	< 0.01

MPI, monthly parasite incidence

The API for 2008 was 207.5/1000 (1136/5475), percentage of *Pf* was 53.3 per cent (606/1136), and ABER was 243.0 per cent (13307/5475). In 2008 there was an increase of 18.7 per cent in API in comparison with 2007 (Table I) The increase in API and ABER during 2008 was statistically significant (*P*<0.01).

## Discussion

Our results showed a large increase in the incidence of malaria in the post tsunami year (2005) and the trend continued till 2008. The geological and environmental changes happened due to tsunami might have contributed to this. The consequent construction activity and labour influx further complicated the malaria situation. A study carried out in the districts of south Andaman in the aftermath of tsunami has highlighted increased salinity of inland water as one of the important risk factor favouring the breeding of *An. sundaicus*^25^.

The other revelation of the study was that of almost doubling of percentage of *Pf* in 2007 and 2008. The increase in incidence of *Pf* coincided with the increased influx of labour from the districts of West Bengal and the beginning of large scale construction activity.

The present study arrived at three important epidemiological deductions. One, the incidence of acute malaria infections (percentage by age) among the stable indigenous Nicobarese population was indicative of high endemicity of malaria in Nancowry group of islands^22^. Two, more than 5 per cent of the tribal children aged < 10 yr presenting with fever turned positive for malaria parasite indicating high transmission setting^26^. Three, the study identified Nancowry group of islands as high risk area for malaria transmission *i.e*., SPR for the past three years was more than 5 per cent, *Pf* proportion was more than 30 per cent, and the tropical aggregation of labour in Nancowry Islands^23^.

Though, the patient register maintained at CHC, Nancowry, showed higher incidence of malaria among migrant labourers than indigenous tribal people but, the mass malaria pasitemia survey showed higher point prevalence of malaria among indigenous tribe. This could be due to low health seeking behaviour of the tribes. This is evidenced by lower proportion of smears examined by passive surveillance at CHC, Nancowry. The findings that 10 per cent of native tribes had asymptomatic parasitemia, and almost 75 per cent of labourers suffered from fever during the past month is a pointer that the workers hailing from non-endemic or moderately endemic settings became victims of malaria in epidemic proportion in high endemic and high transmission setting. They visited CHC, Nancowry and got treated with anti-malarials. This may be the reason for low point prevalence of malaria among migrant labourers in comparison with indigenous tribe.

The SPR for the first week of April 2008 was more than 3SD of the average for past three months indicating the build up of parasite in the community^23^,^24^. Though mass drug administration (MDA) or MRT has limited role in the long term control of malaria, these could have a part to play in the management of epidemics^27^. Moreover, in some cases, it had a marked effect on parasite prevalence and on the incidence of clinical malaria^28^. The short term effect of MRT was dramatic as evidenced by post-intervention parasitemia survey. It had no effect on long term control of malaria.

In India, insecticide impregnated bed nets (IIBNs) with pyrethroids have been tried for malaria control with variable results^29^–^33^. Lambdacyhalothrin IIBNs distributed in an endemic tract of Orissa, showed overall decline in parasite rates among all age groups, while it was well received and accepted by the local tribals of Assam, following which the SPR decreased significantly^30^,^31^. In Nancowry, though the usage of bed net was almost 100 per cent (with the exception that migrant labourers) but it did not help much in containing malaria in this island. This emphasizes the fact that malaria control needs multi-pronged approach.

The limitation of the study was that the entomological aspect was not looked into. Secondly, the study was carried out in a far flung island almost 350 km away from base station, hence there were innate logistical difficulties in covering larger sample of the population for mass fever and malaria parasitemia survey. Higher sample size in post-intervention survey would have helped in analysing the effect of MRT in better way.

In conclusion, there was a deterioration of malaria situation at Nancowry post tsunami. The MRT had dramatic immediate effect on the occurrence of malaria but from long-term perspective it had no effect. To validate the hypothesized reasons for the increased occurrence of malaria following tsunami further studies are required and this will help evolve appropriate intervention strategy.
